# Investigating the Self‐Thinning Rule in Plantation Forests: Analyzing the Relationship Between the Basal Area and Height Growth in Southern China

**DOI:** 10.1002/ece3.71034

**Published:** 2025-03-06

**Authors:** Shisheng Long, Xuefeng He, Siqi Zeng, Huashun Xiao

**Affiliations:** ^1^ Faculty of Forestry Central South University of Forestry and Technology Changsha Hunan China

**Keywords:** basal area, canopy closure, maximum density line, self‐regulatory, self‐thinning

## Abstract

The self‐thinning rule in forest stands is fundamental to the development of density management strategies, as it determines the maximum stand density achievable for a given tree size. Accurate modeling of the maximum density line is crucial, but selecting representative data points for this purpose remains a challenge. Using 18 years of data from five *Cunninghamia lanceolata* plantations with varying initial planting densities, this study investigated whether relationships between mean tree basal area (*g*) and height (*H*) can reveal forest developmental stages and identify when stands begin self‐thinning and reach maximum density. Our results showed a significant linear relationship (*p* < 0.05) between *g* and *H* after self‐thinning was established, supporting the presence of self‐regulatory growth mechanisms. These findings enabled the development of a novel sample selection method for constructing more accurate maximum density line models, outperforming traditional methods that rely on arbitrary thresholds. Additionally, we derived formulas to describe total stand basal area (*G*_1.0_) during different growth stages, revealing positive correlations with mean height during early growth and negative correlations with mean diameter during self‐thinning. This research advances the understanding of self‐thinning dynamics and provides practical tools for improving density management in plantation forestry.

## Graphical abstract

The mean basal area and height showed a significant linear relationship during two growth stages (from canopy closure to prethinning, anaphase self‐thinning). The forest may have a self‐regulatory mechanism that ensures a relative balance of mean basal area and height.

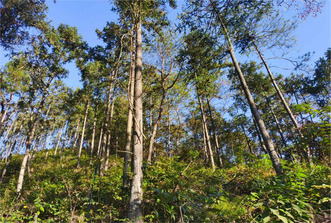

## 1 Introduction

Self‐thinning refers to the natural process by which trees compete for limited resources, such as light, water, and space, leading to the mortality of some individuals when these resources can no longer meet their physiological needs (Pretzsch and Biber 2005; Vospernik and Sterba 2015; Yang and Burkhart 2017). As a newly planted forest matures, the number of trees per unit area reaches a maximum density limit. Maximum density represents the carrying capacity of a forest under specific ecological and site conditions, reflecting the upper limit of stand occupancy as described by maximum density lines (Dong et al. 2024). With continued growth, the forest's capacity to accommodate trees decreases, leading to a decline in maximum density (Zeide 1991; Bi, Wan, and Turvey 2000). The maximum density line is a statistical model that quantifies the relationship between a forest's maximum density and its characteristics. Constructing this line for a specific forest enables more informed decisions regarding forest management and thinning practices (Burkhart 2013). Many researchers have developed models to describe the relationships between maximum forest density and stand variables (Reineke 1933; Yoda, Kira, and Ogawa 1963; Zeide 1987; Li, Wu, and Zou 2000; Burkhart 2013). However, the complexity and variability inherent in the self‐thinning process and model construction continue to pose significant challenges to these efforts.

Reineke (1933) proposed that the maximum stand density (*N*_max_) and mean diameter at breast height (*D*) exhibit a linear relationship in double logarithmic coordinates. He asserted that the slope of the maximum density line for any tree species remains constant at −1.605, independent of age and stand characteristics. However, subsequent studies have shown that the slopes for certain tree species deviate from this value (Zeide 1985; Gadow 1986; Westoby and Howell 1986). Most scholars now agree that the slope varies, with empirical values ranging from −2.33 to −1.54 (West, Brown, and Enquist 1997; Pretzsch and Biber 2005). In their research on herbaceous plants, Yoda, Kira, and Ogawa (1963) reported a relationship between plant size and density similar to Reineke's equation, which led to the well‐known 3/2 rule. Notably, the slope derived from Yoda's 3/2 rule and the West, Brown, and Enquist (WBE) model is −2 (Zhang, Bi, and Jeffrey 2005; Fu et al. 2008). These variations in slope values have fueled ongoing debates regarding the characteristics of the maximum density line models.

One significant factor contributing to differences in slope estimates is sample selection during model development. A robust sample must accurately reflect the maximum density and stand growth under self‐thinning conditions (Weller 1987b). Therefore, identifying when a forest has reached a self‐thinning state is crucial for sample selection. Current methods often rely on visual inspection, mortality rates, canopy closure, and maximum diameter intervals to select appropriate samples (Weller 1987a; Osawa and Sugita 1989; Lonsdale 1990; Bi and Nigel 1997). However, these methods are subjective and can yield inaccurate results, especially when they depend on observer experience. Quantile regression and stochastic frontier methods are also used to construct maximum density lines, but they often prioritize statistical precision over ecological significance (Tian et al. 2020; Long et al. 2022). To date, no universally accepted method exists for selecting samples to accurately model maximum density lines.

In China, basal area–volume tables based on the maximum size–density theory are widely used in forest inventories and management to estimate stand volume relative to stem density per unit area. These tables assume that for fully stocked stands (with a stem density of 1.0), given a certain mean height, the total basal area and volume are constant across different site conditions. One common model used in these tables is $G_{1.0}=a+\mathrm{bH}$, where *G* is the basal area and *H* is the mean height. However, several studies have challenged the validity of this assumption in practical applications (Luo, Zeng, and He 2001). Nevertheless, relationships between various stand variables, such as height and diameter, diameter and age, and basal area and height, are well documented in fully stocked stands (Burkhart 2013; Mensah et al. 2018; Sharma et al. 2019; Ciceu et al. 2020). Nonetheless, the relationship between the mean basal area and mean height in fully stocked stands has yet to be thoroughly investigated. Exploring this relationship may offer valuable insights into the principles governing self‐thinning dynamics.

**FIGURE 1:**
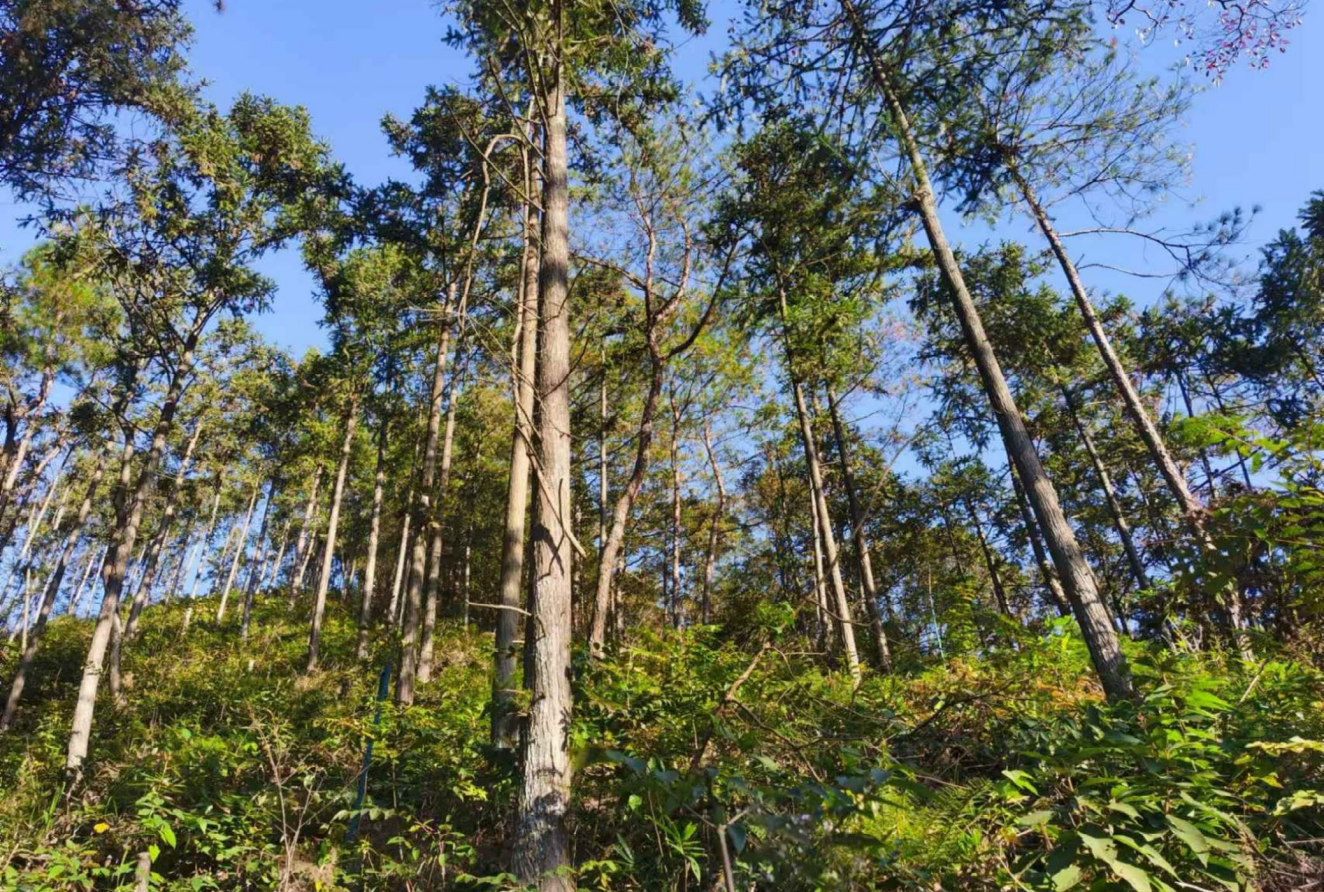
Typical *Cunninghamia lanceolata* plantation forests in southern China.

China's plantation forests span 79.54 million hectares, accounting for 36.15% of the country's total forested area. The southern region of China is a key area for plantation distribution, with major species including *Cunninghamia lanceolata* (Figure 1), *Pinus massoniana* , and *Eucalyptus*. Currently, most of these plantation forests are in their early or middle stages of growth, characterized by high carbon sequestration rates and significant potential for further carbon sink development. Compared with natural forests, plantation forests offer advantages such as simplified forest structure and easier density control. Understanding the maximum density line and self‐thinning rules in plantation forests can inform the development of effective forest management strategies, thereby improving forest quality and enhancing China's contributions to global climate change mitigation.

The objectives of this study, which was conducted in *C. lanceolata* plantation forests in southern China, are as follows: (1) to examine the growth relationship between the mean basal area (*g*) and mean height (*H*) in fully stocked plantation forests; (2) to propose a new method for sample selection in the construction of maximum density lines; and (3) to further validate and refine self‐thinning theory with respect to changes in stand basal area characteristics.

## 2 Materials and Methods

### 2.1 Experimental Sites and Measurements

This study utilized data from *C. lanceolata* collected from experimental plots in southern China. The *C. lanceolate* plots were located in the Nianzhu forest farm, Jiangxi Province, in southeastern China, and were established in 1981 (Tong, Sheng, and Zhang 2002). Five different planting densities were used: 2 × 3 m (A: 1667 stems/ha), 2 × 1.5 m (B: 3333 stems/ha), 2 × 1 m (C: 4983 stems/ha), 1 × 1.5 m (D: 6633 stems/ha), and 1 × 1 m (E: 9967 stems/ha). Each plot covered an area of 20 m × 30 m. Tree height (m) and diameter at breast height (DBH, cm) for all trees were measured annually in winter from 1986 to 1991 and biennially thereafter until 1999. The site index, which measures site productivity on the basis of the height of dominant trees at a specific age, for plots A and B was 16 m, whereas plots C, D, and E had a site index of 14 m. Continuous observations were made over an 18‐year period, and no artificial thinning was performed during this time. A summary of the stand variables for *C. lanceolata* across the five plots is presented in Table 1.

**TABLE 1:** Summary of the stand variables for the five plots of *Cunninghamia lanceolata*.

Stand age (year)	Trees per hectare					Mean tree height (m)					Mean DBH (cm)				
	A	B	C	D	E	A	B	C	D	E	A	B	C	D	E
5	1667	3333	4983	6633	9967	3.7	3.8	3.5	3.4	3.3	5.0	4.8	4.0	3.8	3.5
6	1667	3333	4983	6633	9967	5.0	4.8	4.5	4.4	4.3	7.6	6.5	5.8	5.4	4.9
7	1667	3333	4983	6617	9950	6.3	5.9	5.6	5.2	5.1	9.8	8.1	7.0	6.4	5.8
8	1667	3333	4967	6583	9900	7.4	7.0	6.5	6.2	5.9	10.9	9.0	7.8	7.2	6.4
9	1667	3333	4950	6533	9733	8.4	7.8	7.2	6.9	6.7	12.1	9.9	8.4	7.8	6.9
10	1667	3317	4933	6450	9483	9.4	8.7	7.9	7.7	7.2	13.0	10.6	9.0	8.2	7.2
12	1667	3267	4883	6350	8933	10.8	9.9	9.0	8.8	8.2	14.4	11.5	9.5	9.1	8.0
14	1667	3217	4750	6050	8467	12.0	11.0	9.8	9.7	9.0	15.5	12.4	10.5	9.8	8.6
16	1633	3133	4583	5717	7467	13.0	11.8	11.0	10.6	9.9	16.5	13.1	11.3	10.3	9.3
18	1617	3067	4367	5550	6850	14.1	13.3	12.2	11.7	10.8	17.0	13.6	11.9	10.9	9.9

### 2.2 Construction of the Maximum Density Line

In 1933, Reineke developed a foundational model to describe the relationship between stand density (*N*) and the mean diameter at breast height (DBH, *D*) in fully stocked, even‐aged forests. This relationship is expressed as a power‐law equation:

(1) $$N=kD^{c}$$

By applying a natural logarithmic transformation, this equation can be converted into a linear form:

(2) lnN=clnD+k

where *N* represents the number of trees per hectare, D is the mean DBH, *c* is the slope of the model, *k* is the intercept, and ln denotes the natural logarithm.

To construct the maximum density line, we adopted Reineke's approach, using this transformed linear equation as the basis for analysis. Reineke originally proposed a universal slope value (*c*) of −1.605. However, later studies by researchers such as Yoda and West et al. have demonstrated that the slope may vary depending on tree species and ecological conditions, with some studies reporting values as steep as −2.

This variability highlights the need for a robust analytical method to determine the most suitable slope for our study. To address this, we applied the least squares method to fit the linearized model. This technique minimizes the sum of squared differences between the observed and predicted values of lnN, providing precise estimates of both the slope (*c*) and intercept (*k*). This approach ensured that the constructed maximum density line accurately represents the species‐specific and site‐specific characteristics of the forests analyzed. Additionally, our study explores the relationship between basal area (*g*) and mean tree height (*H*), which offers complementary insights to Reineke's formula. While Reineke's formula focuses on the link between density and DBH, the g–*H* relationship reflects how tree height growth compensates for changes in basal area and density during self‐thinning. This perspective expands the potential variables that can be used in self‐thinning models, contributing to a more comprehensive understanding of stand dynamics and maximum density thresholds.

### 2.3 Selection of Model Samples

#### 2.3.1 Conventional Methods

The selection of model samples is critical for fitting the maximum density line. Several common methods are employed, including the quantification of mortality in successive measurements (Westoby 1987), the selection of points from each interval (Newton 2006), and relative density (Solomon and Zhang 2002).

Mortalities in successive measurements (MSM): In this method, samples are selected on the basis of a fixed mortality rate. Three different mortality thresholds are considered: the onset of mortality, a 10% mortality rate, and a 15% mortality rate.

Interval method (INT): The stand density is divided into several equal intervals, and the sample with the largest diameter within each interval is selected. Three interval sizes were tested: 500, 1000, and 1500 trees.

Relative density (RLD): The relative density of each plot was calculated, and samples were selected if their relative density exceeded a fixed threshold. Three relative density thresholds were tested: 0.70, 0.80, and 0.90.

#### 2.3.2 New Method for Selecting Model Samples

1. Determining the onset of self‐thinning

**FIGURE 2:**
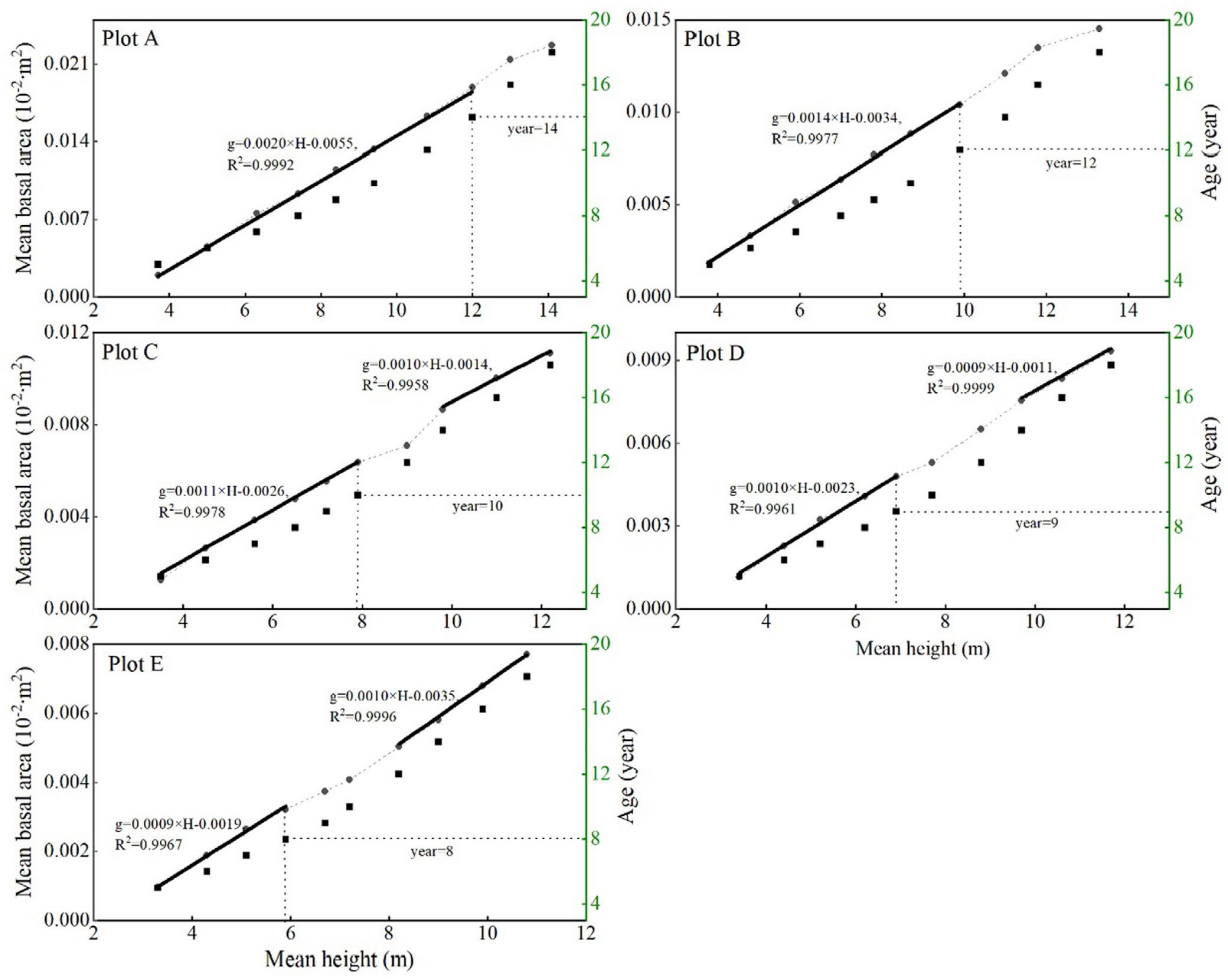
Trends in mean tree height with mean basal area and mean age in sample plots A, B, C, D, and E. The black dots represent the scatter distribution of the mean height versus the mean basal area, the black squares represent the scatter distribution of the mean height versus the mean age, and the black solid line represents the linear model of the mean height versus the mean basal area.

Identifying when self‐thinning begins is challenging, as tree mortality may be due to self‐thinning or environmental factors such as fire, deforestation, or disease. Figure 2 shows a highly significant linear relationship between the mean basal area and mean height for sample plots A, B, C, D, and E up to the ages of 14, 12, 10, 9, and 8 years, respectively. The coefficients of determination for these linear models all exceeded 0.99. This linear relationship breaks down after these ages, likely due to increased competition within the stand, marking the onset of self‐thinning.

To further validate this, Plot C was examined as an example. The linear model for the relationship between the average basal area and average height from years 5 to 9 was *g* = 0.00114 × *H* − 0.00262, predicting an average basal area of 0.0061 m^2^ for year 10, whereas the observed value was 0.0064 m^2^. A one‐sample *t*‐test comparing the residuals from years 5 to 9 with those from year 10 revealed no statistically significant difference (*p* > 0.05). The linear model for years 5–10 was *g* = 0.00114 × *H* − 0.00259, predicting an average basal area of 0.0077 m^2^ for year 12, with an observed value of 0.0071 m^2^. A one‐sample *t*‐test comparing the residuals from years 5 to 10 with those from year 12 revealed a statistically significant difference (*p* < 0.05). These results indicate that the average basal area in year 10 did not deviate from the linear model, whereas the basal area in year 12 did, suggesting that self‐thinning in plot C began after year 10. Similarly, self‐thinning in Plots A, B, D, and E was determined to begin after 14, 12, 10, and 9 years, respectively, on the basis of statistically significant differences in the *t* tests (*t* = −9.105, −9.744, −3.243, and −6.155, respectively; all *p* < 0.05).

2 Major stages of self‐thinning

The self‐thinning process can generally be divided into three stages (Sun et al. 2011): In the early growth stages, all trees have sufficient space, and no mortality is observed. Stage II: In the middle stages, competition for resources intensifies, leading to tree mortality as canopy closure persists. Stage III: In the late stages, the stand reaches its maximum density, where further increases in volume result in corresponding tree deaths.

**FIGURE 3:**
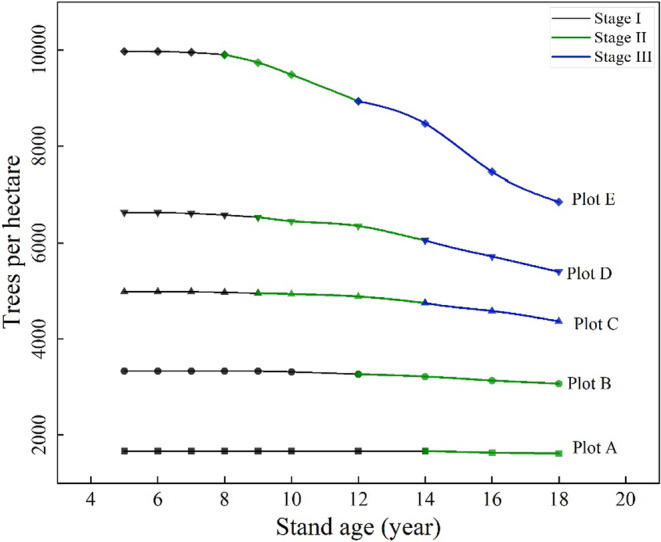
Trends of tree density changes with stand age across self‐thinning stages.

As shown in Figure 2, a significant linear relationship between the mean basal area and mean height was evident during Stage I (from canopy closure to prethinning) across all the sample plots, with coefficients of determination exceeding 0.99. However, in Stage II (early self‐thinning), this linear relationship breaks down, indicating that the stand is undergoing dynamic structural adjustments. During Stage III (late self‐thinning), a strong linear relationship reappears in plots C, D, and E, with coefficients of determination again exceeding 0.99. This relationship was not observed in plots A and B, likely because these plots had not yet entered the third stage of self‐thinning. On the basis of the changes in tree numbers across different plots, as shown in Table 1 and Figure 3, we found that in the age–density relationship, Stage I showed no change in tree number, as tree density remained stable across all plots during this stage. Stage II exhibited irregular changes, with tree density beginning to decline gradually, though the rate of reduction was moderate. In Stage III, a consistent and significant pattern of tree density reduction was observed, particularly in plots C, D, and E. This suggests that Stage II corresponds to the early phase of self‐thinning, where the onset of density reduction and dynamic structural changes becomes apparent. Meanwhile, Stage III represents the phase of maximum stand density adjustment, characterized by a strong linear relationship between basal area and height and consistent density reduction.

3 Selecting the model sample

On the basis of the above analyses, stands in Stage III are typically at maximum stand density. For long‐term monitoring plots, the relationship between the mean basal area and mean tree height should be analyzed. When a plot reaches Stage III, the density and mean diameter at this time can be used as a model samples. These samples, combined from multiple plots, can be used to construct a maximum density line model for the tree species. In the absence of long‐term monitoring data, stem analysis can be performed on average trees from sample plots to determine the growth relationship between basal area and height, thereby obtaining model samples in the same way.

Using this method, we determined that the sample densities and mean DBHs from plots C and D at 14, 16, and 18 years and from plot E at 12, 14, 16, and 18 years could be used as model samples for constructing the maximum density line model.

### 2.4 Evaluation of Maximum Density Line Models

The evaluation of maximum density line models compared the performance of four sample selection methods: mortality successive measurement (MSM), interval method (INT), relative density (RLD), and a newly proposed method based on self‐thinning stages. Using data from *C. lanceolata* , the models were assessed using the coefficient of determination (*R* ^2^) to measure the strength of the linear relationship and the relative root mean square error (RRMSE) to evaluate prediction accuracy.

## 3 Results

### 3.1 Comparison of Sample Selection Methods

**FIGURE 4:**
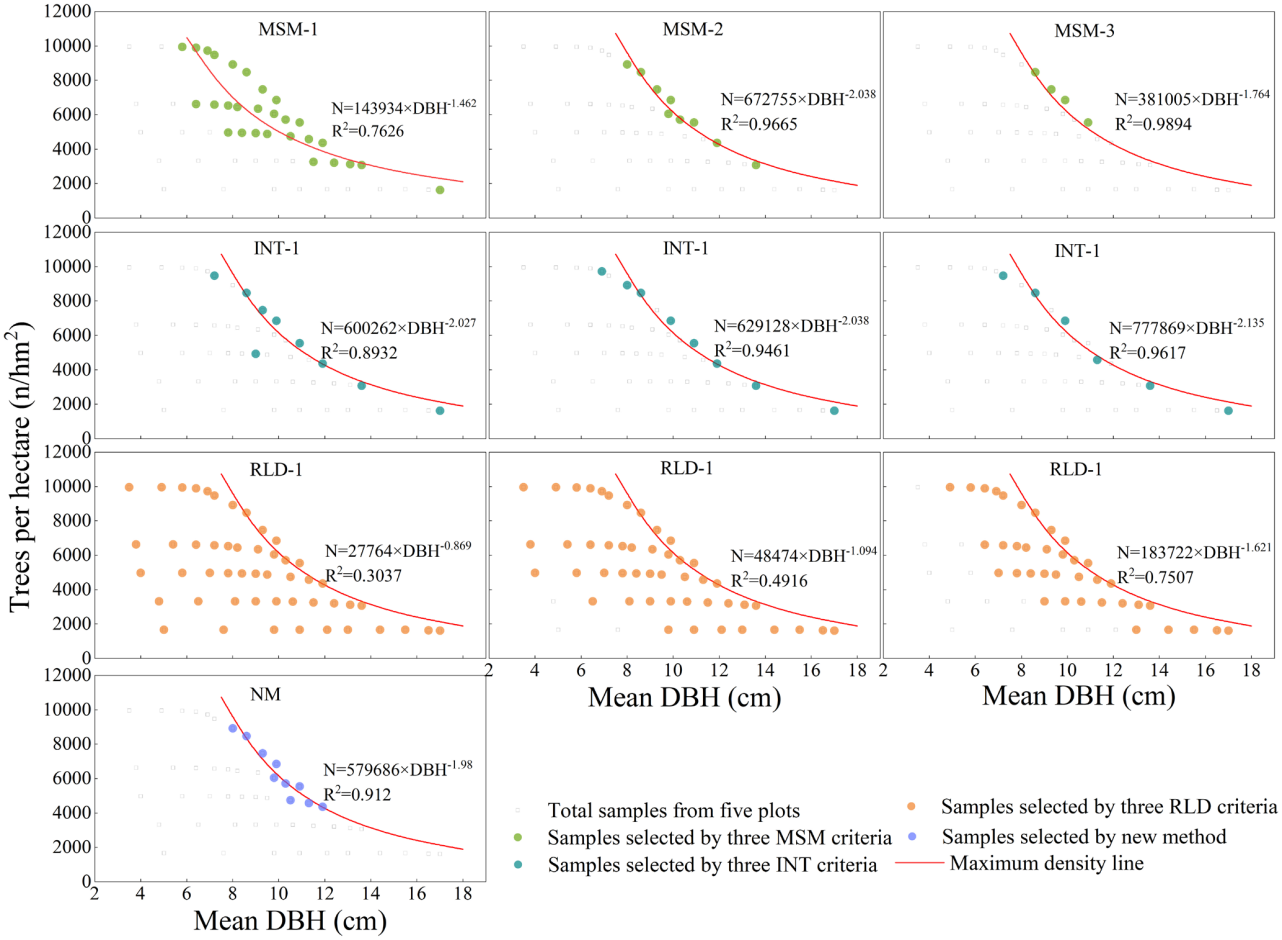
Model samples were selected via four methods: INT, interval; MSM, mortality successive measurement; NM, new method based on self‐thinning stage III; RLD, relative density.

Three conventional sample selection methods (MSM, INT, and RLD), alongside a newly proposed method, were evaluated. Figure 4 shows the results of model sample selection via these approaches. With the MSM method, the number of samples selected decreased as mortality increased, with a high coefficient of variation (75.64%) across mortality criteria. The INT method yielded a reduction in sample size as the strain interval increased, with a smaller coefficient of variation (16.27%). The RLD method also resulted in a decrease in sample number with increasing relative density, with the smallest coefficient of variation (12.44%) across different selection criteria. The new method, however, resulted in no variability in sample numbers, indicating greater consistency. These findings suggest that traditional methods introduce some uncertainty in selecting model samples, whereas the new method provides unique and more reliable sample selection.

### 3.2 Comparison of Maximum Density Line Models

The performance of the maximum density line models derived from the four sample selection methods is summarized in Table 2. For the MSM method, the model based on the 15% mortality criterion achieved the highest coefficient of determination (*R* ^2^ = 0.9894) and the smallest relative root mean square error (RRMSE = 0.25%). However, significant variability was observed in the model slopes and *R* ^2^ values across different mortality criteria, with coefficients of variation of 13.42% and 11.25%, respectively. The INT method exhibited lower variability in both model slopes and *R* ^2^ values (coefficients of variation of 2.36% and 3.14%, respectively) but resulted in higher RRMSEs. The RLD method, on the other hand, showed greater variability (26.39% and 35.56%, respectively) and lower *R* ^2^ values. The new method demonstrated relatively high *R* ^2^ values and relatively low RRMSE values when compared with other sample selection approaches and is not subject to threshold variability. While the MSM 15% mortality achieved the single highest *R* ^2^ and lowest RRMSE, the new method offers significant advantages in terms of consistency and objectivity. Unlike the MSM, INT, and RLD methods, which rely on arbitrary thresholds, the new method objectively selects samples based on the linear relationship between average basal area and average tree height. This eliminates subjectivity in sample selection and ensures reliable performance across varying conditions. Therefore, when considering both consistency and objectivity, the new method provides a robust and transparent alternative for constructing maximum density line models.

**TABLE 2:** Fitting results for the maximum density line model via four methods: MSM (mortality successive measurement), INT (interval), RLD (relative density), and NM (new method).

Methods	Standard	*K*	*c*	*R* ^2^	RRMSE (%)
MSM	Start death	−1.462	11.877	0.7626	2.45
	10% mortality rate	−2.038	13.419	0.9665	0.76
	15% mortality rate	−1.764	12.851	0.9894	0.25
INT	Interval of 500 trees	−2.027	13.305	0.8932	2.28
	Interval of 1000 trees	−2.038	13.352	0.9461	1.81
	Interval of 1500 trees	−2.135	13.564	0.9617	1.75
RLD	RD > 0.70	−0.869	10.232	0.3037	5.95
	RD > 0.80	−1.094	10.789	0.4916	4.92
	RD > 0.90	−1.621	12.121	0.7507	3.30
New method	Stage III	−1.980	13.27	0.9120	0.91

*Note:* Parameters: *K* (model intercept), c (model slope), *R* ^2^ (coefficient of determination), and RRMSE (relative root mean square error).

### 3.3 Derivation of the Total Stand Basal Area

The mean basal area demonstrated a linear relationship with the mean height at both Stages I and III of stand development. On this basis, formulas for calculating the total stand basal area were derived for these stages.

#### 3.3.1 Formula for Total Stand Basal Area at Stage I

As shown in Table 1, the number of trees (*N*) remained constant from canopy closure to pre‐thinning (Stage I). Thus, the total stand basal area (*G*_1.0_) at this stage can be expressed as:

(3) $$G_{1.0}=\mathrm{Ng}$$

where *g* represents the mean basal area. Given the linear relationship between the basal area and tree height (*H*), Equation (3) can be rewritten as:

(4) $$G_{1.0}=\mathrm{Ng}=N\left(a+\mathrm{bH}\right)=a_{1}+b_{1}H$$

where $a_{1}=a∙N$ and $b_{1}=b∙N$ are both constants. This formula mirrors the widely used basal area–volume tables based on maximum size–density theory:

(5) $$G_{1.0}=a+\mathrm{bH}$$

Thus, it can be concluded that the total stand basal area (*G*_1.0_) maintains a linear relationship with height (*H*) from canopy closure to pre‐thinning (Stage I).

#### 3.3.2 Formula for Total Stand Basal Area at Stage III

In Stage III, where self‐thinning occurs, the number of trees (*N*) changes continuously, but the relationship between the basal area and height (*g* and *H*) remains linear. The total stand basal area (*G*_1.0_) during this phase is calculated as:

(6) $$G_{1.0}=Ng=N\left(a+\mathrm{bH}\right)=kD^{c}\left(a+\mathrm{bH}\right)$$

where *k*, *a*, *b*, and *c* are constants. This can be further simplified to:

(7) $$G_{1.0}=kD^{c}\left(a+\mathrm{bH}\right)=D^{c}\left(a_{1}+b_{1}H\right)=\frac{a_{1}+b_{1}H}{D^{C_{1}}}$$

**FIGURE 5:**
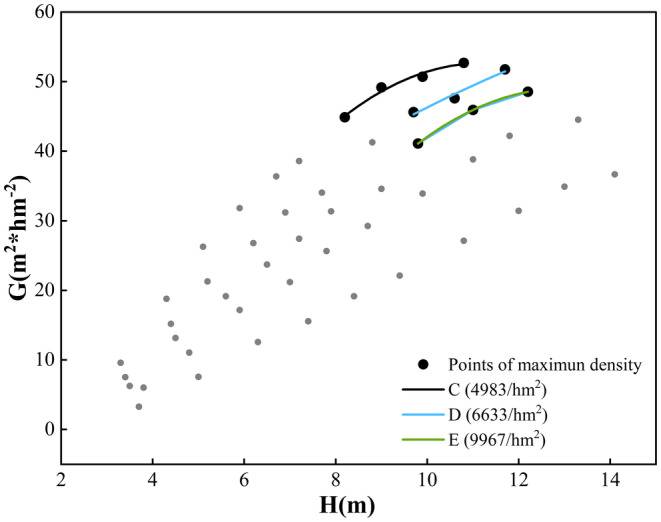
Changes between $G_{1.0}$ and *H* in the maximum stand density at stand C (green lines), stand D (blue lines) and stand E (black lines).

Taking the derivative of *G*_1.0_ with respect to *H* yields:

(8) $$G_{1.0}^{\prime }=\frac{\partial G_{1.0}}{\partial H}=\frac{\partial }{\partial H}\left(\frac{a_{1}+b_{1}H}{D^{c_{1}}}\right)=\frac{b_{1}}{D^{c_{1}}}$$

where *b₁* and *c₁* are constants and *D* represents the mean diameter. As both N and D change throughout self‐thinning, the model slope between *G*_1.0_ and *H* is not fixed. Therefore, the relationship between *G*_1.0_ and *H* during Stage III is not linear. This is further demonstrated in Figure 5, where the slope between *G*_1.0_ and *H* decreases with increasing diameter in stands C, D, and E.

For *Cunninghamia lanceolata* in Stage III, Equation (7) was fitted as:

(9) $$G_{1.0}=\frac{-796.1+210.492H}{D^{1.452}}$$

with an *R* ^2^ of 0.969 and an RRMSE of 1.38%. These results revealed a significant correlation between the basal area (*G*_1.0_), diameter (D), and height (*H*).

In summary, the total basal area (*G*_1.0_) can be expressed as:

(10) $$G_{1.0}=\left\{\begin{array}{c} a+\mathrm{bH}\;\text{Stage}\;I\;\\ \frac{a+\mathrm{bH}}{D^{c}}\;\text{Stage}\;\mathrm{III} \end{array}\right.$$

where *G*_1.0_ is the total basal area, *D* is the mean diameter, *H* is the mean height, and *a* and *b* are model parameters. Stage I covers canopy closure to pre‐thinning, whereas Stage III represents the maximum stand density phase.

## 4 Discussion

The results of this study confirm that forest density plays a crucial role in regulating tree growth, particularly through the self‐thinning process. Higher plant densities were found to reduce the growth space for individual trees, leading to smaller diameters and higher height–diameter ratios, which are consistent with the findings of previous studies (Yamakura 1984; Somchai, Pongsak, and Kyoji 1990; Li, Han, and Wu 2006). The observed decrease in the model slope between the mean basal area (*g*) and height (*H*) with increasing density indicates that as the density increases, the onset of self‐thinning occurs earlier, as predicted by Li and Akio (1999) and Vospernik and Sterba (2015). The linear relationship observed between *g* and *H* during stages I and III of growth suggests that even under varying densities, forests exhibit a self‐regulatory mechanism that maintains a growth balance across different conditions. This finding supports the hypothesis that forests may have inherent systems to adapt to environmental and spatial limitations.

The identification of self‐thinning stages in this study provides new insights into the dynamics of forest growth. The linear relationships between *g* and *H* at both stages I (canopy closure) and III (self‐thinning) highlight the presence of stable growth patterns despite fluctuations in tree density and size. This phenomenon is similar to previous observations in plant systems (Cornelissen et al. 2014; Meyer et al. 2017). Although the duration of the relative balance between *g* and *H* could not be predicted due to the limited observation period, the results suggest that forests naturally maintain a stable growth state until self‐thinning disrupts this equilibrium. This study extends previous findings (White 1987; Zeide 1991; Sun et al. 2011) by demonstrating that, in *C. lanceolata* forests, *g* and *H* remain in balance during self‐thinning, supporting the theory that forests follow self‐regulatory mechanisms across different growth stages.

A comparison of the sample selection methods used to derive the maximum density line models reveals significant differences in accuracy and stability. Traditional methods, such as MSM, INT, and RLD, introduce variability and inconsistencies, whereas the newly proposed method shows higher accuracy, no variability in sample numbers, and more reliable model fitting. This finding aligns with past research that points to variability in slope estimation as a major factor affecting maximum density line models (Osawa and Sugita 1989). The slope of −1.9797 derived from the new method is consistent with that of Yoda and West et al. and further validates the reliability of this approach. This suggests that the new method offers a more objective and accurate means of estimating maximum forest density, especially in plantation forests.

While Reineke (1933) and Yoda, Kira, and Ogawa (1963) proposed that the slope of the maximum density line remains constant across species, this study highlights significant variability in slope on the basis of the site index, density, and species. Previous studies have noted this variability (Zeide 1995; Bi 2001; Pretzsch and Biber 2005), and the findings from this research further emphasize the importance of sample selection in determining slope accuracy. The observed slope variability among the different methods demonstrates that improper sample selection can distort maximum density models. The new sample selection method introduced in this study offers a promising alternative by mitigating these inconsistencies and improving the accuracy of maximum density line models.

The derivation of total stand basal area formulas for Stages I and III offers important insights into the growth dynamics of plantation forests, particularly in relation to self‐thinning. In Stage I, the linear formula $G_{1.0}=a+\mathrm{bH}$ aligns with traditional models but provides a clearer link to the early self‐thinning phase, reflecting stable growth with minimal competition. In contrast, the Stage III formula $G_{1.0}=\frac{a+\mathrm{bH}}{D^{c}}$ introduces nonlinearity due to declining tree numbers and acknowledges the role of diameter in self‐thinning. This approach, which is particularly relevant to plantation forests, enhances the accuracy of predicting stand dynamics and informs more precise thinning practices. Ecologically, these formulas highlight the self‐regulatory mechanisms that forests use to optimize growth under density constraints, offering a more robust framework than previous models that overlooked distinct self‐thinning stages. The improved accuracy of these methods not only benefits plantation management but also contributes valuable insights to forest ecology by revealing adaptive growth strategies across different stages of development.

The results from this study suggest that forests may have the inherent self‐regulatory mechanism that governs the relationship between the mean basal area and mean height. This mechanism ensures that even under changing densities, forests maintain a growth balance at key stages of development. While this self‐regulation was observed in *C. lanceolata* , further research is needed to explore whether this phenomenon exists in other species and forest types. Future studies should focus on obtaining long‐term data to verify the linearity between *g* and *H* across different environments and tree species. Additionally, the applicability of these findings to uneven‐aged forests should be explored to better understand the complexities of forest self‐regulation and its implications for forest management practices.

## 5 Conclusion

This study highlights the critical role of forest density in regulating tree growth through the self‐thinning process. The observed linear relationship between the mean basal area (*g*) and mean height (*H*) during key growth stages supports the presence of self‐regulatory mechanisms that maintain growth balance, even under varying densities. Compared with traditional methods, the new sample selection method introduced in this research demonstrates superior accuracy and stability, offering a more reliable approach for constructing maximum density line models. Additionally, the findings challenge the assumption that the slope of the maximum density line remains constant across species, as significant variability was observed depending on site conditions and density. While this study focused on *C. lanceolata* , further research is needed to explore its dynamics in other species and forest types. The results contribute to the development of effective forest management strategies, particularly in plantation forests, where controlling density is crucial for optimizing growth and maximizing carbon sequestration potential.

## Author Contributions

**Shisheng Long:** conceptualization (lead), formal analysis (lead), methodology (lead), writing – original draft (lead). **Xuefeng He:** methodology (equal), software (equal), writing – review and editing (equal). **Siqi Zeng:** funding acquisition (lead), resources (equal), software (equal), visualization (equal), writing – review and editing (equal). **Huashun Xiao:** data curation (equal), investigation (equal), methodology (equal), project administration (equal), software (equal), validation (equal), writing – review and editing (equal).

## Conflicts of Interest

The authors declare no conflicts of interest.

## Data Availability

The data presented in this study have been uploaded to Dryad (https://datadryad.org/stash/share/J6f_MPDTwUcFVRJFYXDjsHmi6QZ‐J_Mdhn9sQ3M‐oDQ).
